# Chondrogenic Potential of Peripheral Blood Derived Mesenchymal Stem Cells Seeded on Demineralized Cancellous Bone Scaffolds

**DOI:** 10.1038/srep36400

**Published:** 2016-11-08

**Authors:** Shao-Jie Wang, Dong Jiang, Zheng-Zheng Zhang, Ai-Bing Huang, Yan-Song Qi, Hai-Jun Wang, Ji-Ying Zhang, Jia-Kuo Yu

**Affiliations:** 1 Institute of Sports Medicine, Beijing Key Laboratory of Sports Injuries, Peking University Third Hospital, Beijing 100191, China; 2 Department of Joint Surgery, Zhongshan Hospital of Xiamen University, Xiamen, China

## Abstract

As a cell source with large quantity and easy access, peripheral blood mesenchymal stem cells (PBMSCs) were isolated and seeded in porcine demineralized cancellous bone (DCB) scaffolds, cultured in chondrogenic medium and evaluated for *in vitro* chondrogenesis. Bone marrow MSCs (BMMSCs) and articular cartilage chondrocytes (ACCs) underwent the same process as controls. The morphology, viability and proliferation of PBMSCs in DCB scaffolds were similar to those of BMMSCs and ACCs. PBMSCs and BMMSCs showed similar chondrogenesis potential with consistent production of COL 2 and SOX 9 protein and increased *COL 2* and *AGC* mRNA expressions at week 3 but the COL 2 protein production was still less than that of ACCs. Minimal increase of hypertrophic markers was found in all groups. Relatively higher *ALP* and lower *COL 10* mRNA expressions were found in both MSCs groups at week 3 than that in ACCs, whereas no significant difference of *COL 1* and *SOX 9* mRNA and MMP 13 protein was found among all groups. To conclude, PBMSCs shared similar proliferation and chondrogenic potential with BMMSCs in DCB scaffolds and could be an alternative to BMMSCs for cartilage tissue engineering. Further optimization of chondrogenesis system is needed regardless of the promising results.

Cartilage injury due to trauma or articular inflammatory disease is one of the major causes of disability in both developing and developed countries, and has therefore become an increased social and economic burden. The injured cartilage has poor intrinsic regenerative capacity and cell-based tissue engineering present a prospective treatment strategy for cartilage lesion. Autologous chondrocyte implantation (ACI) has been developed to treat cartilage defects since 1987^1^. However, several major concerns have been reported such as donor-site morbidity, limited number of available cells, and rapid dedifferentiation of chondrocytes when expanded *in vitro*^2^,^3^. Hence, alternative cell sources have been explored and the mesenchymal stem cells (MSCs) separated from different tissues were most popular due to their potential of proliferation and differentiation^4^,^5^,^6^. As the most extensively used MSCs, BMMSCs have its disadvantages such as limited available volume and invasiveness.

With the superiority of its less invasion and larger quantity for clinical settings, MSCs from peripheral blood (PBMSCs) have been isolated^7^, and used as an alternative to BMMSCs to repair bone and cartilage regeneration *in vivo*^8^,^9^. In our previous studies, PBMSCs of rats and rabbits were identified and confirmed similar proliferation and multi-lineage differentiation potential compared to BMMSCs^10^,^11^. However, those studies were mostly investigated in two dimensional monolayer culture which is significantly different from natural cell environment. Biological behavior of PBMSCs seeded on 3D porous scaffolds remains largely unknown. An in-depth understanding in this regard will provide more reliable and predicative information for *in vivo* cartilage tissue engineering,

The purpose of the present study was to explore the chondrogenic potential of the PBMSCs in demineralized cancellous bone (DCB), a commonly used 3D bioactive scaffold for cartilage tissue engineering. In this study, xenogeneic DCB scaffolds derived from porcine were utilized due to sufficient size for transplantation and more extensive sources for clinical use comparing to the allograft. The rabbit PBMSCs were seeded on porcine DCB scaffolds and the cell morphology, proliferation, and chondrogenic potential of the PBMSCs were accessed. The BMMSCs and articular cartilage chondrocytes (ACCs) were seeded on the same scaffold as controls (schematic seen in Fig. 1.). We hypothesized that the PBMSCs might show similar results to the controls regarding proliferation and chondrogenic potential in 3D microenvironment.

## Results

### Scaffold gross appearance, micro-morphology, pore sizes and porosity

**Figure 2:**
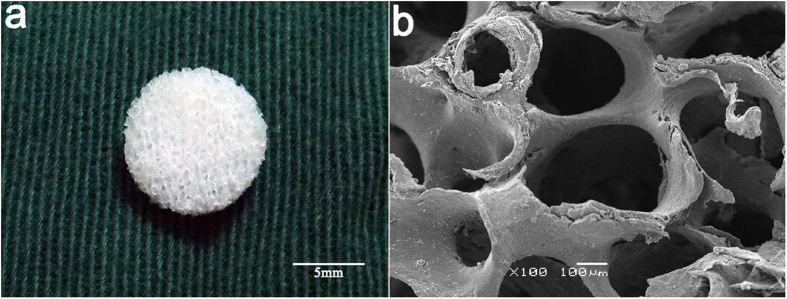
(**a**) Representative macroscopic images of demineralized cancellous bone (DCB) scaffolds (scale bar = 5 mm). (**b**) Typical scanning electron microscopy (SEM) image revealing that DCB scaffolds have a spongy 3D structure with open and interconnected micropores of various pore sizes (scale bar = 100 μm).

A scaffold with 2 mm thickness and 6 mm in diameter was shown in (Fig. 2a). As revealed by SEM, DCB scaffolds exhibited natural porous structures with highly interconnected open pores ranging in size from 137.9–558.1 μm (Fig. 2b). The mean pore size of the DCB scaffolds was 332.3 μm [95% CI (69.6, 86.3)], and the mean porosity of the scaffolds was 77.9 ± 0.03%.

### Cell attachment, distribution and viability

**Figure 3:**
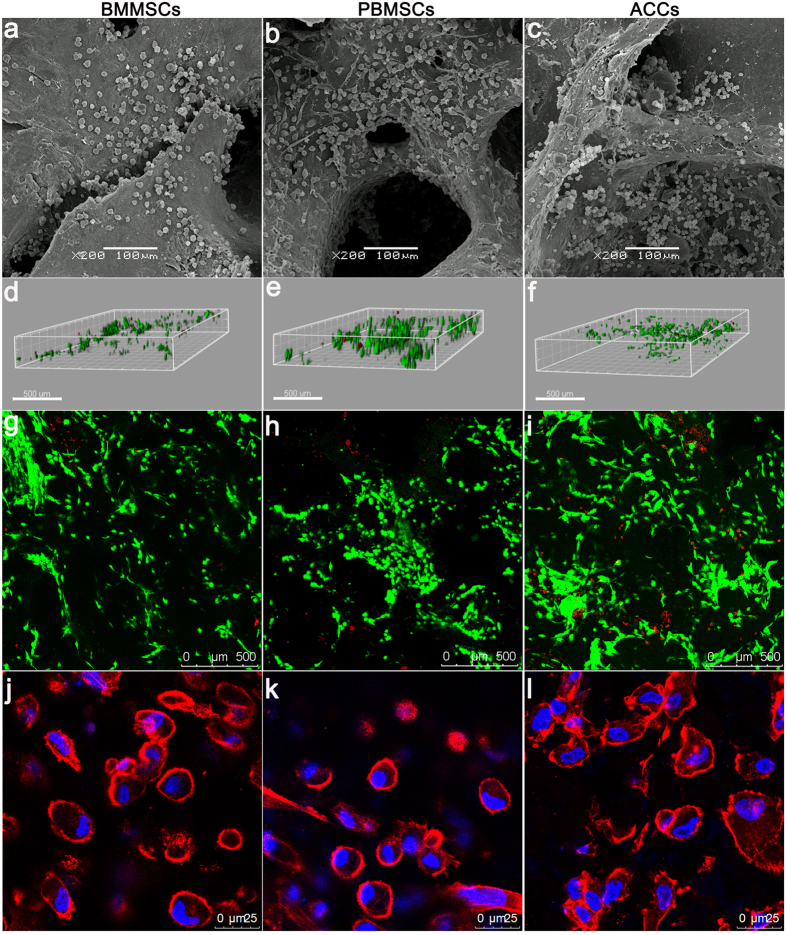
Representative images of attachment, distribution, viability and morphology of BMMSCs, PBMSCs, and ACCs in DCB scaffolds. (**a–c**) Scanning electron micrograph images of scaffolds seeded with different cells showing excellent cell attachment after 24 h of culture. (Scale bar = 100 μm); (**d–f**) 3D renderings of live and dead cells in 3D scaffolds after 72 h of culture (Scale bar = 300 μm). (**g–i**) Confocal microscopic images of Live/Dead staining demonstrated *in vitro* cell proliferation and viability of three groups in 3D scaffolds after 72 h of culture. Red, dead cells; green, live cells (Scale bar = 500 μm). (**j–l**) Immunofluorescent staining revealing cell morphology ((**j**): BMMSCs, k: PBMSCs, (**l**): ACCs) after 1 week of chodrogenic induction culture. (Red, Rhodamine phalloidin stained cytoskeleton; blue, Hoechst stained nuclei, Scale bar = 25 μm).

After 24 h of culture, SEM observation revealed that the cells of all the groups have firmly attached to the surface and micropores, forming interconnections and clusters. Similar to the BMSCs, the PBMSCs showed multiple morphologies with elongated or polygonal shape. The ACCs appeared polygonal or cobblestone-like morphology (Fig. 3a–c).

**Figure 4:**
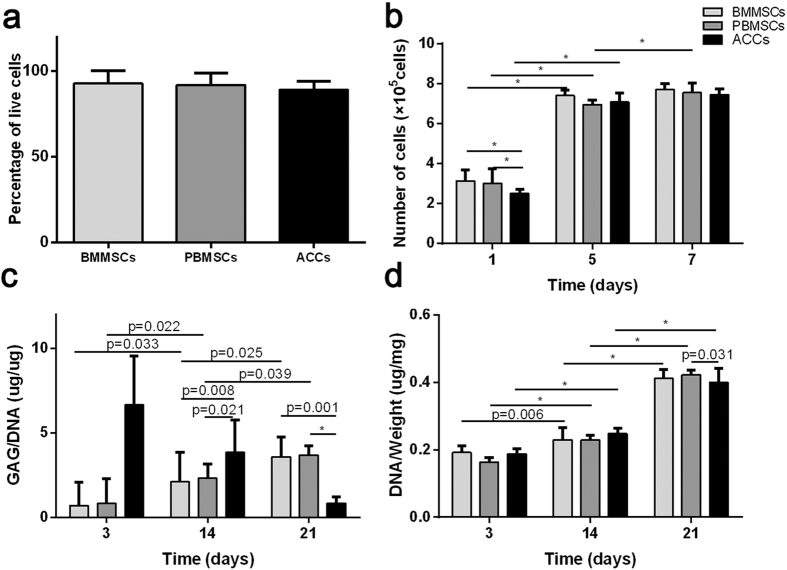
*In vitro* cell proliferation and cartilage-specific matrix production in DCB scaffolds. (**a**) Percentage of live cells detected by LIVE/DEAD staining. (**b**) CCK-8 assay detected that the number of cells in the three groups increased over time. (**c**) Glycosaminoglycan (GAG) deposition in the scaffolds by different populations of cells was assessed by DMMB assay. (**d**) DNA content assessment showed increased DNA contents in various cell-seeded DCB scaffolds at various time points. (Results are expressed as mean ± 95% CI, n = 5, *P < 0.001).

After 72 h of culture in growth medium, majority of seeded cells survived in the scaffolds (Fig. 3d–j). 3D rendering of the cell-seeded scaffolds showed that PBMSCs, BMMSCs and ACCs distributed evenly in both surface and inner part of the DCB scaffold. Although there was a minimal number of dead cells in the PBMSCs group, no significant difference of live cell percentage was found among the three groups (n = 5, *p* > 0.05, Fig. 4a).

After chondrogenic culturing for 1 week, cytoskeleton staining showed that the cells in DCB scaffolds of all groups displayed a similarly rounded shape (Fig. 3j–l). The both MSCs shifted from a fusiform fibroblast-like morphology to a rounded chondrocyte-like shape of groups, indicating the possible phenotype changes upon chondrogenic induction.

### *In vitro* cell proliferation on 3D scaffolds

The Cell Counting Kit-8 assay demonstrated the proliferation of all the three groups during the first week (Fig. 4b). The number of PBMSCs and BMMSCs were initially higher than ACCs group at day 1. Thereafter, no significant difference was found among three groups at day 7 and the results were comparable to those at day 5, except the PBMSC group showed further increased proliferation.

DNA contents of all the three groups further increased during 3 weeks of culture (Fig. 4D). PBMSCs showed relatively lower DNA content at day 3 compared to BMMSCs and ACCs. However, the DNA content of both MSCs groups increased and reached a similar level at day 21, which was significantly higher than that of ACCs group (n = 5, *p* < 0.001).

### Cartilage-specific gene expression analyses

**Figure 5:**
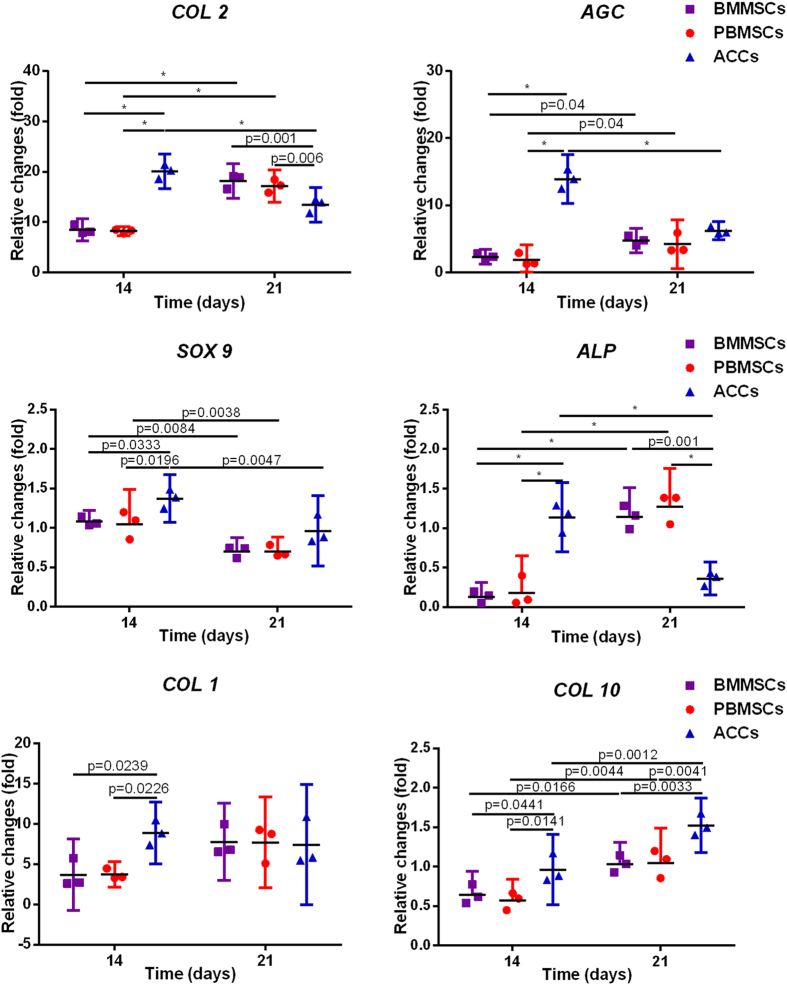
Expression of the cartilage-specific genes, *COL 2, AGC* and *SOX 9*, fibrous-cartilage gene *COL 1* and osteogenesis marker gene *ALP* and *COL 10* within the scaffolds. (Results are expressed as mean ± 95% CI, n = 3, *p < 0.001).

We assessed the hyaline-cartilage specific genes SRY-related high mobility group-box gene 9 (*SOX 9*), aggrecan (*AGC*), collagen type II (*COL 2*), fibrous cartilage-related gene collagen type I (*COL 1*), and osteogenesis marker alkaline phosphatase *(ALP)* and collagen type X *(COL 10)*. Significant differences in gene expression were found between day 14 and day 21 (Fig. 5). For the MSCs-seeded constructs (BMMSCs and PBMSCs), greater up-regulation of *COL 2, ALP* and *COL 10*were observed at day 21 compared to that at day 14 (n = 3, p < 0.05). For the ACCs-seeded constructs, significantly lower expression of *COL 2*, *AGC* and *ALP* was found at day 21 compared to day 14 (n = 3, *p* < 0.05). Despite an increase for both MSCs constructs and a decrease for ACCs constructs, *COL 1* expression in both MSCs and ACCs constructs remained stable after 14 days of culture. *SOX 9* expressions in all groups slightly increased at day 14 and then dropped at day 21 compared to that at day 0, whereas *COL 10* expressions increased after 3 weeks of culture. In addition, the gene expression varies among three cell groups. Expressions of *COL 2, AGC, SOX 9* and *ALP* by PBMSCs and BMMSCs were still significantly lower than those by ACCs at day 14 (n = 3, *p* < 0.05). However, at day 21, *Sox 9* and *AGC* expressions in MSCs-seeded constructs were similar to those in ACCs-seeded constructs (n = 3, *p* > 0.05). Higher *ALP* expression level was found in both MSC groups, while higher *COL 10* in ACCs-seeded constructs (n = 3, p < 0.05). It should be noted that the levels of *ALP* and *COL 10* were minimal with only one-fold increase. In addition, the *COL 1* expression level of both MSCs groups was similar to that in ACCs at week 3 (n = 3, *p* > 0.05).

### Extracellular matrix deposition on 3D scaffolds

GAG synthesis was initially strongest in ACCs seeded scaffold but decreased after 14 days. Both PBMSCs and BMMSCs groups showed continuously increased GAG secretion (Fig. 4C), reaching a similar level at day 14 and a higher level at day 21, compared to that of ACCs group. No significant difference of GAG content was found between PBMSCs and BMMSCs groups at all time points (n = 5, *p* > 0.05).

**Figure 6:**
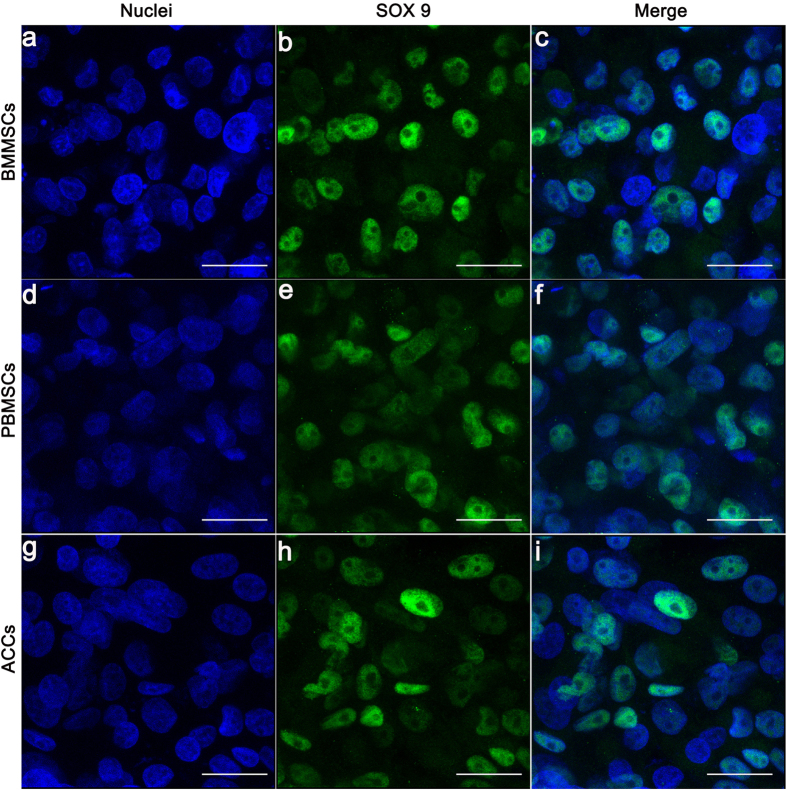
Immunofluorescence analysis of SOX9 expression in the scaffolds seeded with BMMSCs (**a–c**), PBMSCs (**d–f**), and ACCs (**g–i**) after 21 days of *in vitro* chondrogenic differentiation culture (Scale bar = 25 μm).

**Figure 7:**
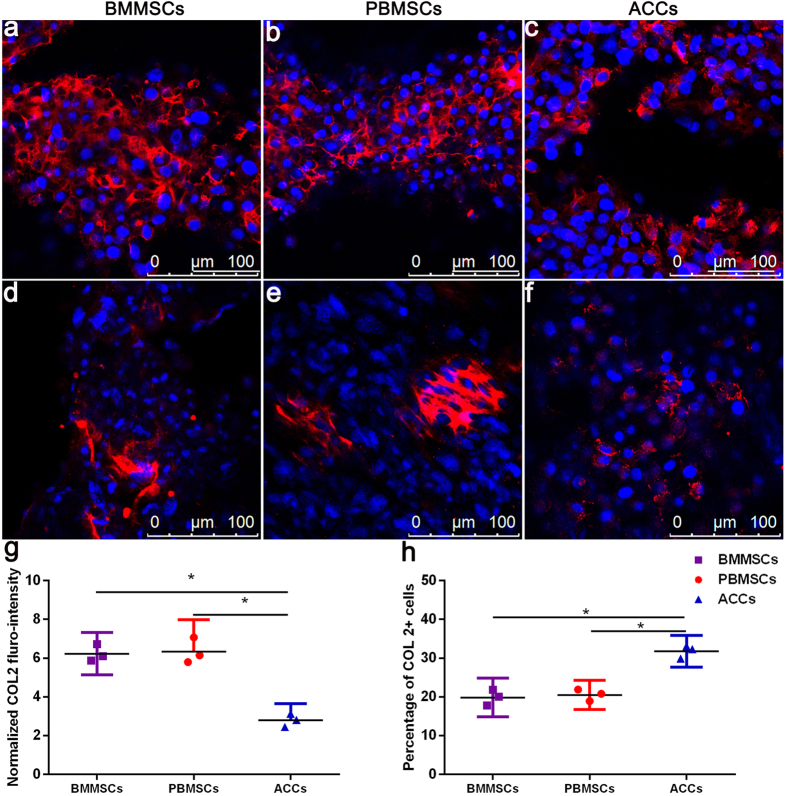
Immunoflurorescent staining of COL 2 in the scaffolds seeded with BMMSCs (**a,b**), PBMSCs (**b,e**), and ACCs (**c,f**) after 21 days of *in vitro* culture. Robust expression of COL 2 was present in three groups of cell-seeded scaffolds ((**a–c**), Scale bar = 100 μm). In a few areas ((**d–f)**, Scale bar = 100 μm), MSCs of partial chondrogenensis produced more COL 2 than hypertrophic ACCs (**g**), while the percentage of MSCs secreting COL 2 is significantly less than that of ACCs (**h**). (Results are expressed as mean ± 95% CI, n = 3, *P < 0.001).

Immunoflurorescence images of the cell-scaffold constructs were shown in Figs 5 and 6. SOX 9 displayed nuclear localization and COL 2 was detected as an extracellular signal. After 3 weeks of *in vitro* chondrogenic culture, diffuse and robust extracellular signal around MSCs were observed, which was similar to that in the ACCs group. Majority of cells in three groups showed nuclear expression of SOX9 (Fig. 6). Strong COL 2 expression was detected for all the three groups (Fig. 7a–c). But in some confined areas, the distribution of COL 2 was uneven and scattered (Fig. 7d–f). COL 2 expression by MSCs in these areas is stronger than that in ACCs group (Fig. 7g). In terms of the percentage of COL 2 positive cells, the ACCs group showed significantly higher level than that that of the MSCs groups (Fig. 7h). These cells with limited expression of COL 2 could be a result of partially differentiated MSCs or hypertrophic ACCs or MSCs.

**Figure 8:**
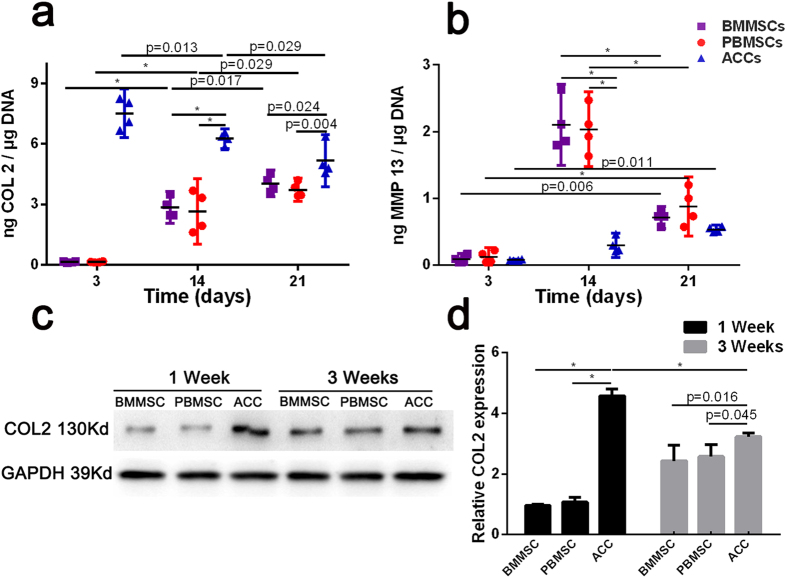
Quantification of COL 2 and MMP 13 secretion of cell-seeded scaffolds cultured under chondrogenic conditions. (**a,b**) Concentration of COL 2 and MMP 13 in culture media of cell-scaffold constructs were determined by ELISA (n = 4, *p < 0.001). Data were normalized to the DNA content of cell-scaffold constructs; (**c**,**d**) Western blotting analysis of COL 2 produced by cell-seeded scaffolds undergoing chondrogenesis culture. (Results are expressed as mean ± 95% CI, n = 3, *P < 0.001).

ELISA analysis was used to quantitatively evaluate the extracellular protein produced by the cells in constructs. As shown in Fig. 8a, content of COL 2 in the culture medium of both MSCs groups increased over a 3-week period of *in vitro* culture. The COL 2 content in the culture medium of the ACCs group gradually decreased but was still significantly higher than that in both MSCs groups at week 3. Meanwhile, hypertrophic marker matrix metallopeptidase 13 (MMP 13) in the culture medium increased over time in all groups; the level of MMP 13 peaked at week 2 in both MSCs groups, but subsequently decreased to a level similar to that in the ACCs group at week 3 (Fig. 8b). The expression of COL 2 was also evaluated by Western blotting analysis of the cell-scaffolds lysate (Fig. 8c,d). Both MSCs groups secreted markedly increased content of COL 2 after 3 weeks of chondrogenic differentiation, while COL 2 production by ACCs groups decreased in the same period. Consistent with the ELISA results, the COL 2 production was higher in ACCs group than that in MSCs groups at all time points.

## Discussion

Loss of cartilage remains an intractable and challenging issue for clinicians. Among a number of therapeutic strategies for promoting cartilage repair, tissue engineering may offer great promise for the regeneration of damaged cartilage. For clinical practice, compared to BMMSCs, PBMSCs present unique advantages such as less invasion, easier access, and larger quantity, in comparison with BMMSCs. In the present study, we compared the survival and chondrogenic ability of PBMSCs to those of BMMSCs and ACCs in 3D scaffolds. The results of the present study found that **a)** PBMSCs displayed good cell viability and proliferation potential in a 3D environment; **b)** PBMSCs could differentiate into chondrocyte-like cells and form cartilage-specific matrix in 3D scaffolds with minimal expression of hyperthrophic markers; **c**) PBMSCs exhibited similar viability and chondrogenic potential in DCB scaffolds compared to BMMSCs; **d)** COL 2 production of PBMSCs or BMMSCs increased with time but was still less than that of early passage ACCs in DCB scaffolds. To our knowledge, this study is the first attempt to elaborate the *in vitro* cellular activity of PBMSCs on xenogenic DCB scaffolds in comparison with that of BMMSCs and ACCs.

According to SEM observation, three groups of cells readily attached and spread on DCB surface and inner pores. In addition, a uniform cell distribution pattern of PBMSCs with few dead cells was observed with confocal microscopy after *in vitro* culture for three days, which revealed high cell viability in 3D scaffolds. Moreover, a typical morphological change from a fibroblast-like to a rounded chondrocyte-like shape occurred after MSCs upon chondrogenesis induction, suggesting the MSCs acquiring chondrocyte phenotypes. Notably, CCK-8 and DNA content assays detected gradually increased number of PBMSCs, indicating continued proliferation of PBMSCs and the biocompatibility with the DCB scaffolds.

Differentiation of MSCs into chondrocytes is associated with expression of cartilage-specific genes. These genes include cartilage extracellular matrix genes, such as *COL 2*, *AGC*, and the transcription factor *SOX 9*, a key regulator of chondrogenic differentiation. After being seeded in DCB scaffolds and cultured in chondrogenic condition over 3 weeks, MSCs from both PB and BM expressed increased *COL 2* and *AGC* mRNA. At the protein level, the nuclear expression of SOX 9 and extracellular deposition of COL 2 were visualized by immunoflurorescent imaging across the scaffolds. The increased expression of COL 2 protein over time by MSCs-seeded scaffolds was quantitatively confirmed by ELISA analysis of the culture media and Western blotting analysis of cell-scaffold lysate. The abundant expression of SOX9, COL 2, and GAG confirmed the chondrogenic differentiation of the PBMSCs in 3D scaffolds.

As shown in the results, the mRNA expression of the *COL 2* was found to be higher in both MSC-seeded constructs than that in ACCs-seeded constructs at day 21. In contrast, the protein level of COL 2 was lower in MSCs-seeded scaffolds than that in ACCs group. In general, protein expression level is in line with the gene expression level. The reason of the discrepancies between mRNA and protein expression in our study might be due to the posttranscriptional regulation of the mRNA which has been reported previously^12^. For instance, microRNAs were known to regulate the target mRNA translation either through translational silencing of the mRNA or degradation of the mRNA in osteoarthritis and chondrogenesis^13^,^14^. It should be noted that there was a tendency of increased COL 2 production of both MSCs with time, thus it is reasonable to expect the same even higher level of COL 2 of MSCs groups compared to that of ACCs with longer culturing time or *in vivo* transplantation.

In the present study, chondrogenic PBMSCs secreted similar amount of cartilage ECM compared to BMMSCs, which provided an *in vitro* evidence of our previous finding in a rabbit model that PBMSCs and BMMSCs shared the same cartilage repair capacity^10^,^11^. 3D cell culture systems can more accurately mimic the actual *in vivo* microenvironment than 2D cultures^15^,^16^. Cellular behaviors in DCB scaffolds are thus more reflective of *in vivo* cellular bioactivity. The reason for the similar chondrogenisis of MSCs from PB and BM might be that PBMSCs and BMMSCs shared the same cell origin, the bone marrow^17^. However, chondrogenesis of MSCs varies in different 3D scaffolds^18^,^19^, thus additional comparative studies are needed to evaluate chondrogenic capacity of PBMSCs in other 3D scaffolds besides DCB.

In addition to cartilage-related components, the gene expression analysis showed increased *ALP, COL 10* and *COL 1* during 3 weeks of culture, and ELISA analysis also detected increased MMP 13 level in MSCs- and ACCs-seeded scaffolds. In the meantime, the expression of SOX 9 was down-regulated at day 21. These results indicated the differentiated MSCs and ACCs underwent terminal differentiation (hypertrophy) after long time culture, which was also reported in previous studies^20^. The chondrogenically differentiated MSCs have been reported to have the tendency to differentiate towards a hypertrophic phenotype, leading to induction of hypertrophic and osteogenic markers, such as MMP 13, ALP, and COL 1^21^. As shown in Fig. 7, in some confined areas, the differentiated MSCs were observed to scattered but relative more COL 2 than ACCs. These areas presented a scenario during *in vitro* culture where some MSCs are either not completely differentiated or becoming hyperthrophic, whereas some ACCs are losing chondrocyte phenotypes. However, the levels of *ALP, COL 10* and *COL 1* found in our study were relatively low compared to chondrogenic markers *COL 2* and *AGC*. The increased MMP 13 in MSC-seeded scaffolds peaked at day 14 and decreased thereafter. It has been reported that MMP 13 play a role in physiological matrix remodeling at growth plate^22^, and MMP-13 is constitutively produced in human chondrocytes^23^. Therefore, considering minimal increase level of hyperthrophic markers during 3 weeks’ culture, majority of MSCs and ACCs seeded in DCB scaffolds still maintained chondrocyte phenotypes.

Notably, differentiated MSCs produced less COL 2 than monolayer expanded ACCs did. This was consistent with previous studies which revealed differentiated MSCs expressed lower levels of COL 2 and higher levels of hypertrophic markers compared to chondrocytes^24^,^25^. In contrast, some studies found the opposed evidence that the pre-differentiated MSC-seeded scaffolds produced enhanced or similar levels of COL 2 or AGC than chondroctyes^26^,^27^,^28^,^29^. The difference might be due to varied passage of the chondrocytes used in those studies. Primary or passages 1–2 chondrocytes were used in the studies showing superiority of chondrocytes, whereas osteoarthritic or long term expanded chondrocytes were used in studies favoring induced MSCs. According to the above data, the COL 2 production of the differentiated PBMSCs might be similar to that of osteoarthritic or *in vitro* expanded chondrocytes. But it is still difficult to produce MSCs-derived chondrocytes similar to primary or the early passage ACCs under the current differentiation conditions in the present study. However, *in vitro* simulation mimicking biochemical and biomechanical conditions of the native joints has been confirmed to promote the proliferation and chondrogenic differentiation of MSCs^30^,^31^, which should be further studied in the future.

Additionally, the increased fibrocartilage and osteogenic markers could be partially attributed to the scaffold material property itself, which was believed to affect resident cells behaviors such as adhesion, growth and differentiation^32^. COL 1 is an organic component of bones and a component of fibrous cartilage, and has been shown to stimulate bone formation^33^,^34^. The natural DCB scaffolds used in the present study were derived from natural decellular and demineralized bone matrix and are mainly composed of COL 1. This property of the scaffold might have mediated the increased expression of *COL 1* and *AL*P by resident cells (MSCs or ACCs), as have been reported by other studies using DCB scaffolds^35^,^36^,^37^,^38^. Therefore, maintaining chondrogenesis and preventing hypertrophy of MSCs and ACCs during *in vitro* culture still represent a challenge in cell-based therapies. Fortunately, there have been studies showing that hydrogels prepared from natural or synthetic polymers can mimic natural microenvironment of articular cartilage, and promote chondrogenic differentiation by encapsulated cells^39^,^40^,^41^. With these advances, the chondrogenic conditions and biocompatible scaffold can be tuned for optimal cell adhesion and proliferation, nutrient diffusion, and growth factor delivery to reduce hypertrophy and hopefully achieve hyaline cartilage matrix production.

In the present *in vitro* study, xenogeneic DCB scaffolds derived from porcine were utilized due to sufficient size for transplantation and more extensive sources for clinical use comparing to the allograft. DCB mainly contains COL 1 and does not elicit immunogenic reaction, as the antigenic surface structure of DCB is destroyed during demineralization^42^. The excellent biocompatibility between three cell populations and xenogeneic DCB scaffolds indicated that xenogeneic DCB scaffold is a safe and bioactive material for tissue engineering and would offer substantial benefits such as comprehensive availability and large quantities.

There are still some limitations for the present study. Firstly, the DCB scaffold used in this study was a bioactive scaffold with biological interaction with the seeded cells, which might influence the proliferation and the chondrogenic potential. So it is not appropriate to generalize the results to all the scaffolds. The chondrogenic ability of the PBMSCs in the chemical synthetic 3D porous scaffolds is still unknown. Secondly, the outcome of the *in vivo* transplantation was still unknown in spite of the favorable result of the adhesion, proliferation and chondrogenesis result in the present *in vitro* study. More studies should be needed in the future.

## Conclusions

The PBMSCs successfully underwent chondrogenesis in the 3D porous xenogeneic DCB scaffolds under chondrogenic conditions. No significant difference was found between the PBMSCs and the BMMSCs in terms of the adhesion, proliferation and extracellular matrix production. PBMSCs might be an alternative to BMMSCs for cartilage tissue engineering, considering the extra advantages of absence of donor site morbidity, easy availability of PB and large quantity. Further optimization of the *in vitro* chondrogenesis culture system is needed to yield chondrogenic MSCs more similar to the native chondrocytes.

## Materials and Methods

### Isolation and culture of MSCs and ACCs

All animal experimental protocols were approved by the Animal Care and Use Committee of Peking University and the methods in the current work were carried out in accordance with the Guide for the Care and Use of Laboratory Animals. Two-month-old adult New Zealand White rabbits weighing approximately 1.5 kg were used for isolation of MSCs and ACCs. BMMSC was obtained exactly as our previous report, the isolation, culture, and identification of MSCs derived from PB were performed with modification^11^. Rabbit mononuclear cells (MNCs) derived from PB was obtained after mobilization by granulocyte colony stimulating factor (G-CSF, Qilu Pharmaceutical Co. Ltd.) and CXCR4 antagonist AMD3100. Blood (20 mL) was collected into a sterile heparinized tube from the central artery of the ear. Each blood sample was diluted 1:1 with phosphate-buffered saline (PBS) and then loaded into an equivalent volume of 1.077 g/mL Ficoll-Paque^TM^ Premium solution (GE healthcare, Sweden). The MNC fraction was obtained by gradient centrifugation at 2500 rpm for 30 min and was then resuspended in complete α-MEM with 10% fetal bovine serum (FBS) at a density of 1 × 10^6 ^cells/mL. Complete medium was refreshed every 2 to 3 days. MSCs from BM or PB at passage 5 were used for subsequent experiments.

Chondrocytes were harvested from the knee and shoulder joints of the rabbits. First, the minced cartilage samples were digested for 6 h in 10 mL of 0.2% w/v collagenase type 2 (Gibco BRL Co. Ltd.) solutions at 37 °C. The obtained cell suspension was centrifuged and resuspended in low-glucose DMEM supplemented with 10% FBS (HyClone^TM^, Thermo Scientific, Australia) and 1% penicillin and streptomycin. Isolated chondrocytes were cultured in monolayer cultures in a humidified atmosphere at 37 °C, 5% CO_2_ and 21% O_2_. Passage 2 chondrocytes were utilized for experiments.

### Fabrication of DCB scaffolds

Cancellous bone was obtained from the distal femur and proximal tibia of pigs. The surrounding soft tissues of the bone segments were removed and discarded. Parallel bone resection by an oscillating saw was employed to produce 5-mm-thick bone pieces. These bone pieces were subjected to the following procedures: (1) demineralization in 0.6 mol/L hydrochloric acid at 4 °C for 7 days; (2) defatting in 1:1 (vol/vol) methanol/chloroform solution for 48 h; and (3) deproteinization with 3% hydrogen peroxide for 4 h. The samples were freeze-dried, sterilized with 25 kGy of Co60, and then trimmed into cylinders 2 mm thick and 6 mm in diameter.

### Porosity measurements

Scaffold porosity was measured with a liquid displacement technique. Ethanol was used as the substitution liquid for easy penetration into the pores of the DCB scaffolds. In brief, the DCB scaffolds were immersed in a graduated cylinder containing 15 mL of ethanol for 10 min until ethanol penetrated the pores of the scaffolds. The total volume in the graduated cylinder was recorded as V_1_ mL. Then the drenched scaffolds were removed, and the remaining volume of ethanol was recorded as V_2_ mL. Porosity was calculated by equation (1).


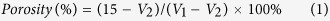


### Cell seeding and cell-scaffold construct culture

DCB scaffolds (2 mm thick and 6 mm in diameter) were carefully seeded with 40 μL of concentrated cell solution (5.0 × 105 cells) using a centrifugal method, as previously reported^43^. The cell-seeded scaffolds were incubated for 2 h to allow initial cell attachment before the addition of 1 ml of fresh medium. Both the PBMSC- and the BMMSC-seeded scaffolds were fed with chondrogenic differentiation medium (RASMX-90041; Cyagen Biosciences Inc., Guangzhou, China) after 3 days of culture in growth medium, and ACC-seeded scaffolds were cultured in low-glucose DMEM with 10% FBS for 3 days and then maintained in the same chondrogenic induction media as used for the MSCs. The culture medium was changed every 2 to 3 days until the cell-scaffold constructs were sent for subsequent analyses.

### Cell distribution, morphology and viability on scaffolds

The characteristics of the scaffolds and cell morphology were observed under Scanning electron microscopy (SEM). Cell-scaffold constructs after 24 h of culture and non-seeded scaffolds were fixed immediately in a mixture of 4 mL 25% glutaraldehyde and 96 mL 10 mmol/L Tris-HCl (pH 7.4), and then dehydrated with a gradient ethanol. Critical point drying was performed in liquid CO_2_ at 37 °C. The specimens were vacuum-coated with a 5-nm layer of gold in a high-vacuum gold spatter coater and then viewed by a scanning electron microscope (S-2500; Hitachi High-Technologies Co, Hitachi-Naka City, Japan). Non-seeded scaffolds were cut and sputter coated with gold and analyzed with SEM. Three scaffolds were included and the size of ten pores for each scaffold was determined by using Image-pro Plus software 6.0 (Media Cybernetics).

The assessment of cell viability in the scaffolds was evaluated using a LIVE/DEAD Viability/Cytotoxicity Kit assay (Invitrogen, Carlsbad, CA, USA). The scaffolds seeded with MSCs or ACCs were cultured in respective growth medium for 72 h. Then, the scaffolds were incubated in 4% paraformaldehyde for 30 min. Each scaffold was immersed in 250 μL of PBS with 2 mM calcein AM and 4 mM ethidium homodimer-1 reagents before incubation for 1 h at room temperature. Excitation wavelength of 568 or 488 nm was used to detect the fluorescence of ethidium homodimer-1 (dead cells = red) or calcein AM (live cells = green). Non-seeded scaffolds were also stained as blank controls to avoid scaffold background effects.

To visualize the live and dead cells in the scaffolds, the volume data were used to create 3D renderings of the cell-seeded scaffolds using Imaris software (7.4.2; Bitplane).

The morphology of the cells in the constructs cultured in chondrogenic induction medium for 1 week were also observed by confocal microscopy. Briefly, the scaffolds were washed with PBS and fixed with 4% paraformaldehyde for 30 min. After penetrating the cell membrane with 1% Triton X-100, the nuclei were stained with Hoechst33258 working solution (Beyotime, Jiangsu, China) for 10 min. The cytoskeleton was stained by rhodamine phalloidin (100 nM; Cytoskeleton Inc., Denver, CO, USA) for 30 min at 37 °C.

### Biochemical analysis for Cell proliferation

The metabolic activity of cells was quantified using a Cell Counting Kit-8 assay (Dojindo Laboratories, Kumamoto, Japan), following the manufacturer’s instructions. At each time point, the cell-seeded scaffolds were thoroughly washed in PBS and then submersed in 10 μL of CCK-8 working solution with 90 μL of fresh medium at 37 °C for 2 h. For the subtraction of the background from the medium, wells were filled with 10 μL of CCK-8 working solution and 90 μL of fresh medium only. The optical density was then observed at 450 nm using a plate reader. The cell content was normalized with each standard curve of cells.

Proteoglycan content was estimated from the glycosaminoglycan (GAG) content using a 1, 9-dimethylmethylene blue (DMMB; Sigma, St. Louis, MO, USA) dye-binding assay to quantify cartilaginous matrix production by cells within the scaffolds. Total GAG was normalized to scaffold wet weight and total DNA content. Briefly, 20 μL of sample was mixed with 200 μL of DMMB reagent, and absorbance was read at 525 nm. A standard curve was established from chondroitin-6-sulfate from shark (Sigma, St. Louis, MO, USA) to compare absorbance for the samples.

The DNA content was measured using a fluorometric assay. The specimens were weighed and then digested in a prepared papain solution (containing 0.5 M EDTA, 0.05 Mcysteine-HCl, and 1 mg/mL papain enzyme) (Sigma) at 70 °C for 48 h. Aliquots of the sample digestion were stained at 37 °C for 20 min with 200 μL of Hoechst33258 working solution (2 μg/mL). The fluorescence intensities were then detected at 360 nm for excitation and 460 nm for emission. The DNA content was normalized with a standard curve of calf thymus DNA (Sigma, St Louis, Missouri, USA).

### Cartilage-specific gene expression analysis

At various time points, samples were removed from culture, briefly rinsed with PBS, and minced into small pieces. Total RNA was extracted using TRIzol reagent (Invitrogen, Carlsbad, CA, USA). Isolated RNA was reverse-transcribed using the MMLV Reverse kit (Promega, Madison, WI, USA), and real-time RT-PCR analysis was performed using an ABI 7300 real-time PCR system (Applied Biosystems, Foster City, CA, USA) with SYBR Green PCR Master Mix (Toyobo, Osaka, Japan). The real-time PCR conditions were as follows: 95 °C for 7 min, followed by 40 cycles of 95 °C for 5 s and 60 °C for 30 s. The disassociation curve showed no nonspecific amplification. The relative expression changes in these target genes were quantified by normalizing their expression to that of the housekeeping gene glyceraldehyde-3-phosphate dehydrogenase (*GAPDH*). Relative gene expression was compared to that at day 0, the values were plotted as 2^−ΔΔCT^. The PCR primers are listed in Table 1.

### Quantification of GAG and DNA content

Proteoglycan content was estimated from the glycosaminoglycan (GAG) content using a 1, 9-dimethylmethylene blue (DMMB; Sigma, St. Louis, MO, USA) dye-binding assay to quantify cartilaginous matrix production by cells within the scaffolds. Total GAG was normalized to scaffold wet weight and total DNA content. Briefly, 20 μL of sample was mixed with 200 μL of DMMB reagent, and absorbance was read at 525 nm. A standard curve was established from chondroitin-6-sulfate from shark (Sigma, St. Louis, MO, USA) to compare absorbance for the samples.

The DNA content was measured using a fluorometric assay. The specimens were weighed and then digested in a prepared papain solution (containing 0.5 M EDTA, 0.05 Mcysteine-HCl, and 1 mg/mL papain enzyme) (Sigma) at 70 °C for 48 h. Aliquots of the sample digestion were stained at 37 °C for 20 min with 200 μL of Hoechst33258 working solution (2 μg/mL). The fluorescence intensities were then detected at 360 nm for excitation and 460 nm for emission. The DNA content was normalized with a standard curve of calf thymus DNA (Sigma, St Louis, Missouri, USA).

### COL 2 and SOX 9 production analysis by immunofluorescence

The production of COL 2 and SOX 9 proteins in cell-DCB constructs were visualized through immunofluorescence after 3 weeks of chodnrogenic culture. Briefly, constructs were fixed with 4% paraformaldehyde for 30 min and then rinsed with PBS. Then the constructs were incubated with 10% fetal bovine serum (FBS) for 60 min at 37 °C followed by incubation with mouse anti-collagen type 2 primary antibody (Calbiochem, Boston, MA, USA) or rabbit anti-SOX9 antibody (Abcam,ab185230,1:2000), for 24 h at 4 °C. After through washing with PBS, the samples were incubated with Alexa Fluor^®^ 594 goat anti-mouse or Alexa Fluor^®^ 488 donkey anti-rabbit IgG antibodies (Invitrogen, Life Technologies) for 1 h at room temperature. The nuclei were counterstained with Hoechst 33258 for confocal microscopy imaging analysis. In the areas where the intensity of COL 2 fluroresence was not evenly distributed, the amount of COL 2 was quantitatively assess expressed by calculating and normalizing the fluorescence intensity of COL 2 to the number of the cells in the immunofluorescence images. The proportion of cells expressing COL 2 was calculated using the Image-Pro Plus software (6.0; Media Cybernetics).

### ELISA analysis of COL 2 and MMP 13

The culture medium of cell-DCB constructs was collected at the designated time points. Supernatant was separated from insoluble residues by centrifugation at 12000 rpm for 10 min. Rabbit MMP 13 and COL 2 ELISA Kits (Cloud-Clone, Corp., Houston, TX, USA) were used to measure the COL 2 and MMP 13 in the culture medium conditioned by the cell-DCB constructs. The assay was carried out according to the manufacturer’s instructions. The COL 2 and MMP 13 concentrations were normalized to DNA content which was determined fluorimetrically using Hoechst staining as previously described.

### Western blotting analysis

The cell-seeded scaffolds were harvested at the indicated time points and rinsed in cold PBS (4 °C) before being lysed in RIPA buffer (Beyotime, Jiangsu, China) supplemented with complete protease inhibitor cocktail (Roche Applied Science, Indianapolis, IN, USA). Protein concentration was measured by BCA protein assay kit (Pierce Biotechnology, Rockford, IL) using bovine serum albumin as the standard. Proteins were run on 10% SDS polyacrylamide gels and electrotransferred to PVDF membrane (Hybond-P, GE Healthcare, Buckinghamshire, UK) at 4 °C for 2 h. The bands were probed with anti-COL 2 (Calbiochem, Boston, MA, USA) at 1:400 dilutions overnight at 4 °C. The proteins were detected by Immobilon Western HRP Substrate (Millipore Corporation, Billerica, MA, USA). *GAPDH* was used as an internal control.

### Statistical analysis

All values are expressed as mean ± 95% confidence intervals (CI) and represent at least three independent experiments. Evaluations of COL 2 fluorescence intensity and percentage of live cells were performed using a one-way analysis of variance (ANOVA); the other data was analyzed using a two-way ANOVA test. P-values < 0.05 were considered significant. When ANOVA results were significant, post-hoc analysis was performed via Tukey’s multiple comparison test. All analyses were carried out using GraphPad Prism version 6.00 for Windows (GraphPad Software, San Diego, CA).

## Additional Information

**How to cite this article**: Wang, S.-J. *et al*. Chondrogenic Potential of Peripheral Blood Derived Mesenchymal Stem Cells Seeded on Demineralized Cancellous Bone Scaffolds. *Sci. Rep*. **6**, 36400; doi: 10.1038/srep36400 (2016).

**Publisher’s note:** Springer Nature remains neutral with regard to jurisdictional claims in published maps and institutional affiliations.

## Figures and Tables

**Figure 1:**
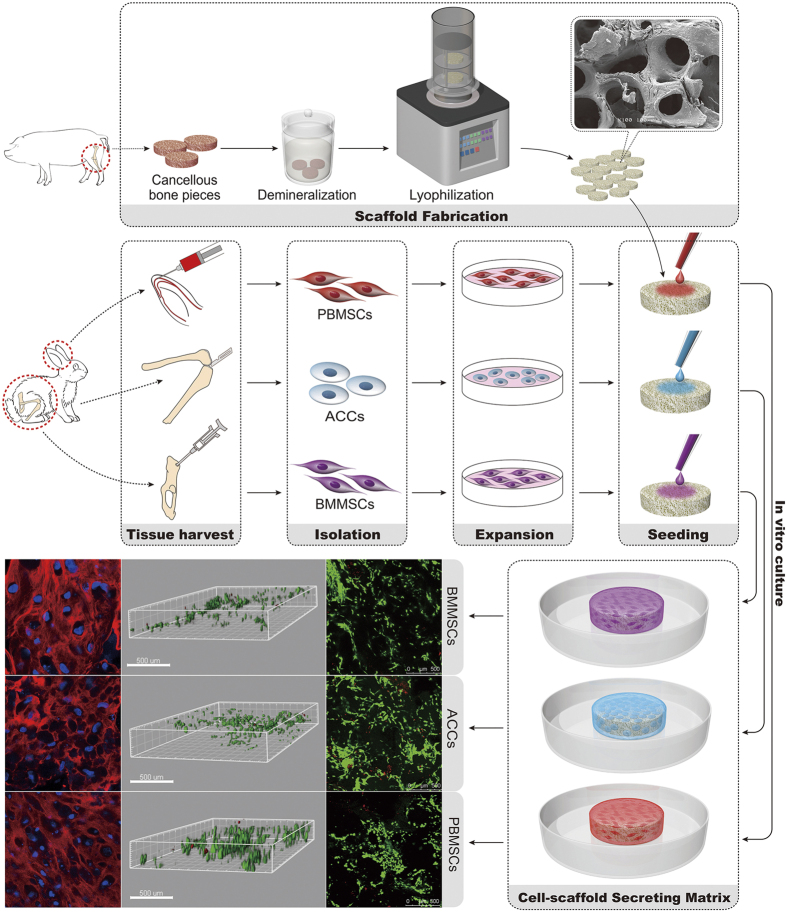
Schematic of the general concept and strategy for this study. BMMSCs, PBMSCs and ACCs were isolated and expanded *in vitro*. The porcine-derived DCB scaffolds were seeded with three groups of cells for *in vitro* culture and subsequent analysis.

**Table 1:** Primer sequences used for real-time PCR.

	Forward primers (5′-3′)	Reverse primers (5′-3′)
*COL 1*	TGGCAAGAACGGAGATGACG	GCACCATCCAAACCACTGAA
*COL 2*	CCACGCTCAAGTCCCTCAAC	AGTCACCGCTCTTCCACTCG
*COL 10*	AAGTGGACCGAAAGGAGACA	TGGAAACCCATTCTCACCTC
*SOX 9*	*AGTAC CGCACC TGCACA AC*	*CGCTTCTCGCTCTCGTTC AG*
*ACG*	*CGTGGTCTGGACAGGTGCTA*	*GGTTGGGGTAGAGGTAGACG*
*ALP*	*CGACACGGACAAGAAACCCT*	*TGTTGTGAGCGTAGTCCACC*
*GAPDH*	*CCATCACCATCTTCCAGGAG*	*GATGATGACCCTTTTGGCTC*

*COL 1:* collagen type I; *COL 2:* collagen type II; *COL 10:* collagen type X; *SOX 9*: SRY-related high mobility group-box gene 9; *AGC*: aggrecan; *ALP*: alkaline phosphatase; *GAPDH*: glyceraldehyde-3-phosphate dehydrogenase.
